# Clinicopathologic correlations of superficial esophageal adenocarcinoma in endoscopic submucosal dissection specimens

**DOI:** 10.1186/s13000-021-01169-1

**Published:** 2021-11-27

**Authors:** Sadhna Dhingra, Firas Bahdi, Sarah B. May, Mohamed O. Othman

**Affiliations:** 1 Formerly, Department of Pathology and Immunology, Baylor College of Medicine, One Baylor Plaza, BCM 315, Houston, Texas 77030. Currently, ProPath Laboratories, River Bend Drive, Dallas, TX 75247 USA; 2 Margaret M. and Albert B. Alkek Department of Medicine, Baylor College of Medicine, McNair Campus 7200 Cambridge Street 8th Floor, Houston, TX 77030 USA; 3 Division of Gastroenterology, Baylor College of Medicine, Margaret M. and Albert B. Alkek Department of Medicine, McNair Campus 7200 Cambridge Street 8th Floor, Houston, TX 77030 USA

**Keywords:** Barrett’s esophagus, Mucosal adenocarcinoma, Submucosal adenocarcinoma, Endoscopic submucosal dissection, Tumor budding

## Abstract

**Background:**

Endoscopic submucosal dissection (ESD) is a novel endoscopic treatment for early esophageal adenocarcinoma (EAC). The western pathologists’ experience with ESD specimens remains limited. This study aimed to correlate histopathologic features of Barrett’s esophagus (BE)-associated adenocarcinoma in ESD resections with clinical outcomes to determine whether they aid future management decisions.

**Methods:**

We retrospectively evaluated 49 consecutive ESD resection specimens from 42 patients with BE-associated adenocarcinoma (24 intramucosal and 18 submucosal EAC) at a single tertiary referral center. Pathologic evaluation included presence of dysplasia, invasive adenocarcinoma, peritumoral inflammation, desmoplasia, lymphovascular and perineural invasion; tumor differentiation, depth of invasion, morphology, and budding; and margin status for dysplasia or carcinoma. Follow up data included endoscopic biopsies in 35 patients and pathology reports of esophagectomies in 11 patients. Poor outcomes were defined as recurrence or residual invasive adenocarcinoma at esophagectomy, metastasis on imaging, or R1 resection in patients undergoing ESD for tumor debulking.

**Results:**

Two patients (8%) with intramucosal adenocarcinoma and 9 patients (50%) with submucosal adenocarcinoma had poor outcomes. Histopathologic features associated with poor outcomes included poor differentiation, lymphovascular invasion, submucosal invasion > 500 μm, tumor budding, and tubuloinfiltrative histologic pattern. Four patients had positive deep margin away from the deepest tumor invasion and did not show residual tumor on follow up.

**Conclusions:**

Our results validated European Society of Gastroenterology (ESGE) guidelines of high-risk pathologic features for additional therapy in esophageal adenocarcinoma and identified tumor budding frequently in association with other high-risk features. Positive deep margin distant from deepest tumor invasion could be procedural and warrants endoscopic correlation for management.

## Introduction

Recent advances in endoscopic resection have led to a paradigm shift in the management of Barrett’s esophagus (BE)–related superficial adenocarcinoma from major surgical resection (esophagectomy), with its high morbidity, to organ-sparing, minimally invasive endoscopic approaches. Endoscopic resection encompasses 2 types of resection: endoscopic mucosal resection (EMR) and endoscopic submucosal dissection (ESD). EMR is useful for en bloc removal of smaller mucosal lesions, but its technical limitations lead to piecemeal resection of larger (> 1.5 cm) lesions. By contrast, ESD permits en bloc resection of larger and deeper lesions and is thus considered superior to EMR for accurate pathologic assessment because of the improved ease of handling and assessing larger en bloc specimens. Histologic assessment of endoscopic resection specimens provides information for risk stratification, which determines further management with surveillance endoscopy or additional therapies, such as endoscopic ablative therapies or endoscopic resections, radical esophagectomy, or chemoradiation.

EMR, coupled with ablative therapies like radiofrequency ablation (RFA), is quite effective for intramucosal adenocarcinoma in BE, and initial studies revealed no superiority of ESD over EMR for these tumors [1]. Recently, ESD has gained momentum in the United States for removing nodular lesions representing dysplasia or early esophageal adenocarcinoma. However, data regarding pathologic evaluation of ESD resection for esophageal adenocarcinoma are sparse. Kumarasinghe et al. [2, 3] published recommendations for optimal handling, processing, and pathologic reporting of ESD specimens. The European Society of Gastrointestinal Endoscopy (ESGE) published guidelines regarding the role of ESD in BE-associated adenocarcinoma and provided recommendations for additional therapeutic management based on histologic findings of ESD resection specimens, which predict the risk of lymph node metastasis [4].

The aim of this study is to evaluate the histopathologic features of BE-associated adenocarcinoma in ESD resections and correlate these features with clinical outcomes and pathology from follow-up esophagectomy (performed according to ESGE guidelines) to examine the robustness of ESGE recommendations for additional therapy.

## Materials and methods

### Study population

Institutional Review Board approval was received for this study. We identified 52 patients with BE who underwent ESD for nodular lesions of early esophageal adenocarcinoma as possible study participants. All patients underwent ESD at the Division of Gastroenterology and Hepatology, Department of Medicine at Baylor College of Medicine and Baylor St Luke’s Medical Center, Houston, TX, between 2015 and 2019. Patients whose final pathology revealed polypoid BE without dysplasia (*n* = 8) and nodular high-grade dysplasia (*n* = 2) were excluded. Thus, 42 patients with ESD specimens showing BE with invasive adenocarcinoma on final pathologic examination were included in this study. As part of routine clinical management, patients were referred for additional therapy (e.g. esophagectomy, chemoradiation) based on pathology of the ESD resection specimens, in accordance with ESGE recommendations.

### Specimen handling and evaluation

**Fig. 1:**
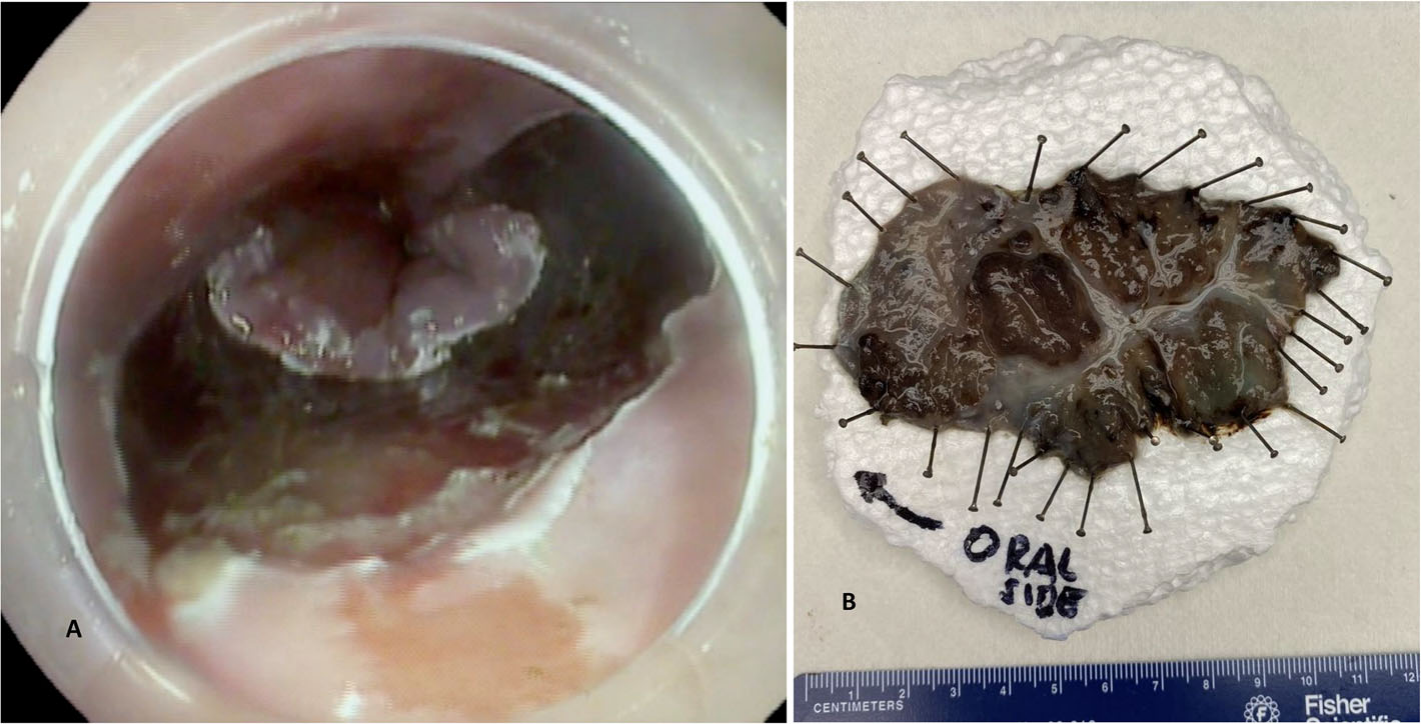
**A**. Endoscopic image of post esophageal endoscopic submucosal dissection. **B**. Gross image of the endoscopic submucosal dissection specimen

All procedures were performed by one endoscopist (MO), who was proficient in both EMR and ESD before study initiation (Fig. 1A). Immediately after resection, each ESD specimen was laid flat and pinned onto a Styrofoam board in the endoscopy suite. The gastroenterologist labeled the orientation “oral end” or “gastric end,” when this may not be apparent (usually the squamous mucosa helped identify the esophageal/oral end) (Fig. 1B). All specimens were subsequently transported immediately to the pathology laboratory, where they were processed based on the published guidelines for handling ESD specimens [2, 3].

For initial microscopic evaluation, only one 5-μm thick, hematoxylin and eosin–stained section was prepared per block. Three deeper levels were obtained for sections exhibiting invasive adenocarcinoma to evaluate maximum depth of invasion (DOI), lymphovascular invasion (LVI), and margin involvement. Immunohistochemical stains were performed when necessary.

### Data collection

Demographic data were collected from patient electronic medical records. Endoscopy reports were reviewed to collect data regarding BE presence and length, gross lesion characteristics, lesion size at endoscopy, resection size at endoscopy, and number of ESD procedures. Recorded outcomes included presence of BE, dysplasia, or carcinoma on follow-up endoscopic biopsies, pathology of follow-up esophagectomy, and presence of biopsy-proven distant metastasis or metastasis on imaging. Duration from ESD to last follow-up was recorded**.** Good outcome was defined as curative resection (R0 resection), disease locally cured with follow-up endoscopic resection and/or RFA, subsequent esophagectomy without residual invasive adenocarcinoma, and no imaging evidence of metastasis. R0 resection was defined as; mucosal and deep margins negative for invasive adenocarcinoma but not necessarily negative for high-grade dysplasia. Poor outcome was defined as recurrent or residual invasive adenocarcinoma on follow-up esophagectomy; metastasis on imaging, with or without biopsy confirmation; or R1 resection in patients with known esophageal adenocarcinoma treated with chemoradiation who were considered poor surgical candidates and underwent ESD for tumor debulking.

### Microscopic evaluation

**Fig. 2:**
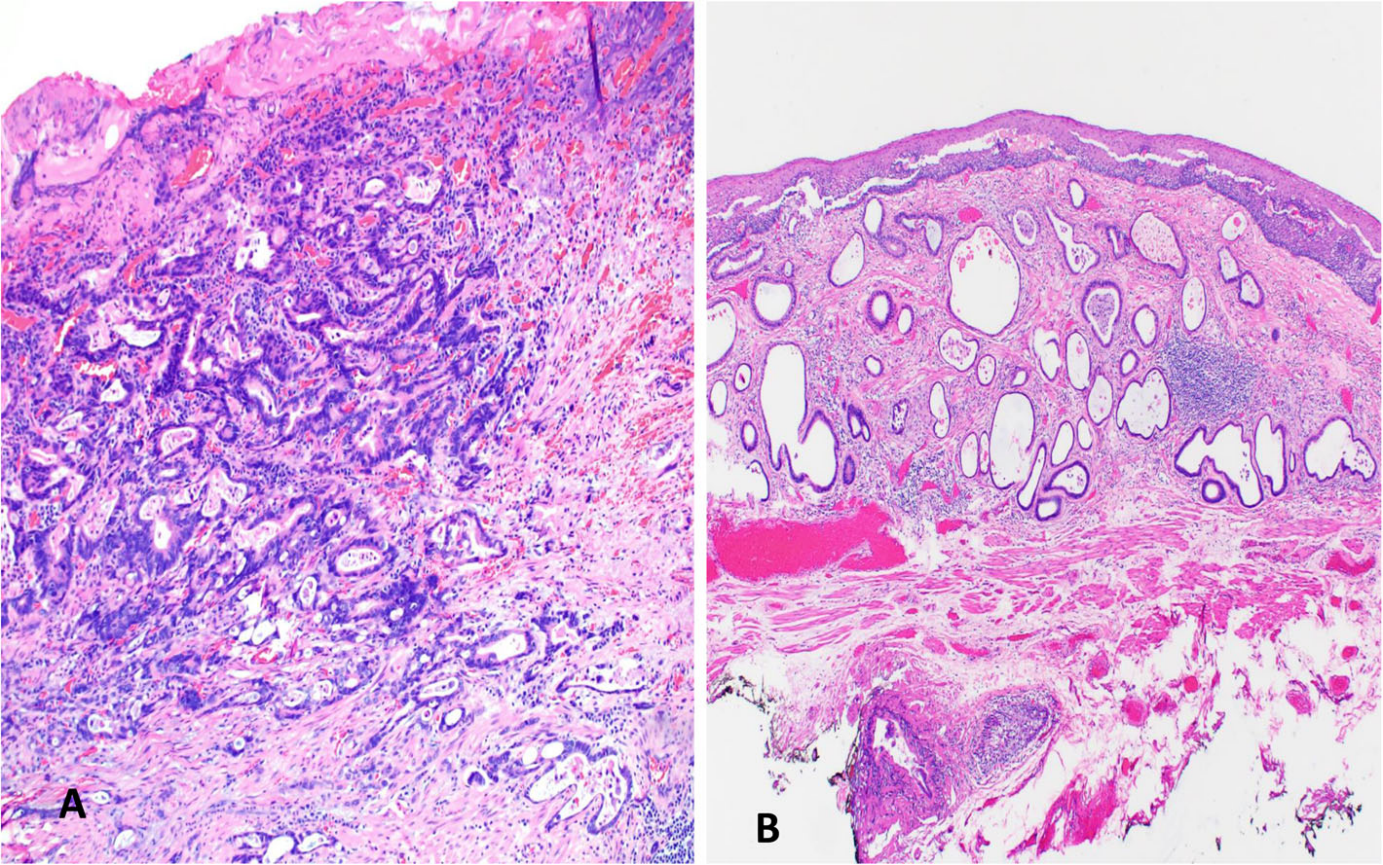
**A**. Invasive adenocarcinoma with tubuloinfiltrative pattern. Hematoxylin and Eosin stain. × 100. **B**. Invasive adenocarcinoma with tubulocystic pattern. Hematoxylin and Eosin stain. × 40

All slides were reviewed by one gastrointestinal pathologist (SD) who evaluated the presence of intestinal metaplasia; muscularis mucosae duplication; low-grade dysplasia, high-grade dysplasia, or invasive adenocarcinoma; and tumor multifocality. Invasive adenocarcinoma histologic pattern was recorded as tubular, papillary, mucinous, or signet ring cell type. Tumor with mixed patterns were evaluated for percent component of high-risk patterns such as mucinous or signet ring cell change. Tubular pattern was subclassified as tubuloinfiltrative, characterized by small tubules and glands with an infiltrative pattern (Fig. 2A), or tubulocystic, characterized by dilated or microcystic tubules with rounded borders, some of which exhibited intraglandular papillae or signet ring cells (Fig. 2B). Other recorded histologic features were tumor DOI, differentiation, and budding; peritumoral inflammation; and presence of desmoplasia, LVI, large-vessel invasion, perineural invasion, and dysplasia or carcinoma at peripheral and deep margins. In oriented specimens, mucosal margin positivity was reported in relation to proximal esophageal, distal gastric, left lateral, or right lateral margins. Margin was considered “positive” when tumor was present at the margin.

**Fig. 3:**
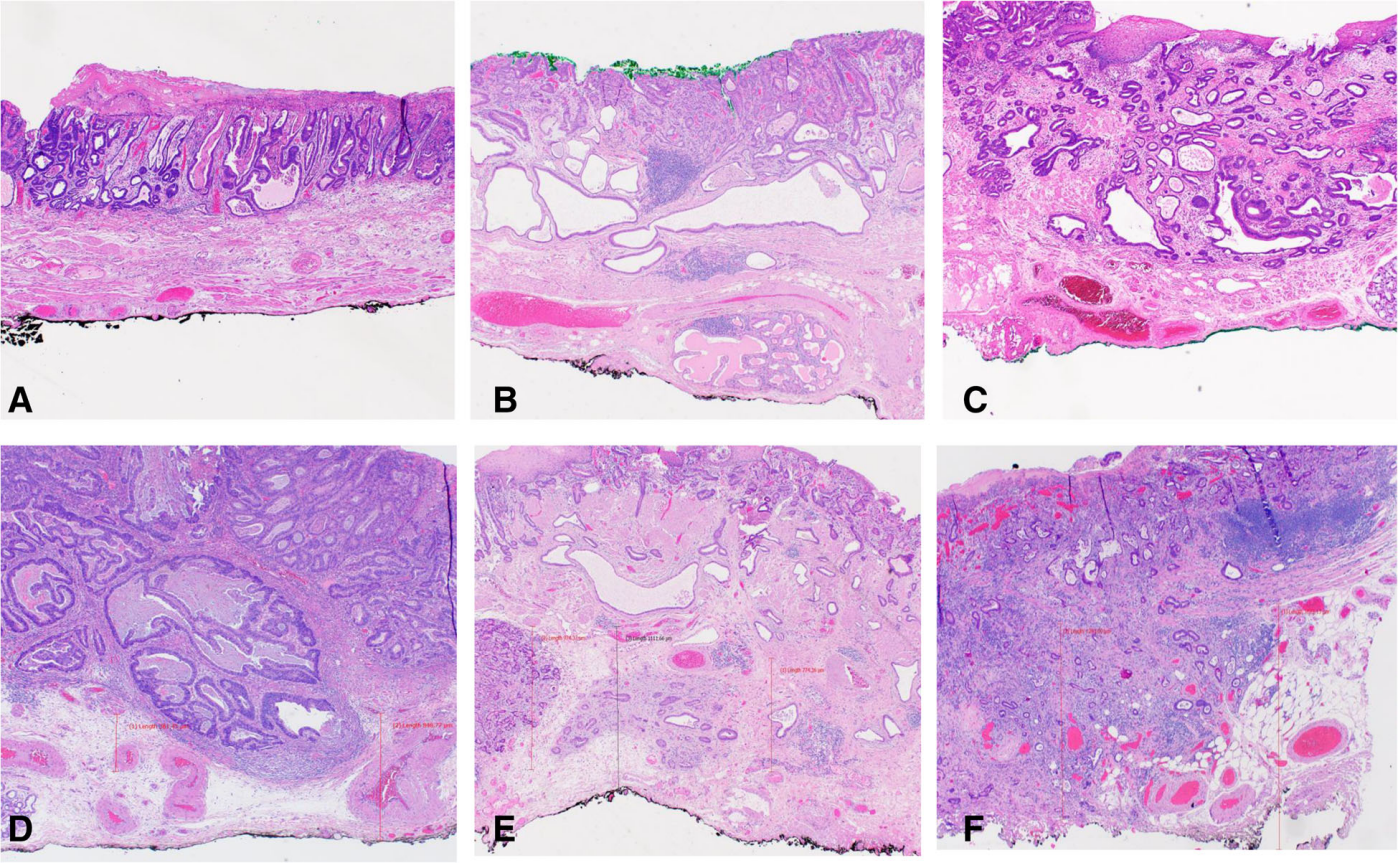
**A**. Invasive intramucosal adenocarcinoma infiltrating into the superficial layer of muscularis mucosae, Vieth and Stolte DOI: m2. **B**. Invasive intramucosal adenocarcinoma infiltrating into layer between superficial and deep muscularis mucosae, Vieth and Stolte DOI: m3. **C**. Invasive intramucosal adenocarcinoma infiltrating into the deep layer of muscularis mucosae, Vieth and Stolte DOI: m4. **D**. Invasive intramucosal adenocarcinoma infiltrating into the superficial submucosa ≤ 500 μm, Vieth and Stolte DOI: sm1. **E**. Invasive intramucosal adenocarcinoma infiltrating into submucosa to a depth between 500 to 1000 μm, Vieth and Stolte DOI: sm2. **F**. Invasive intramucosal adenocarcinoma infiltrating into deep submucosa > 1000 μm, Vieth and Stolte DOI: sm3. Hematoxylin and Eosin stain. × 40

**Fig. 4:**
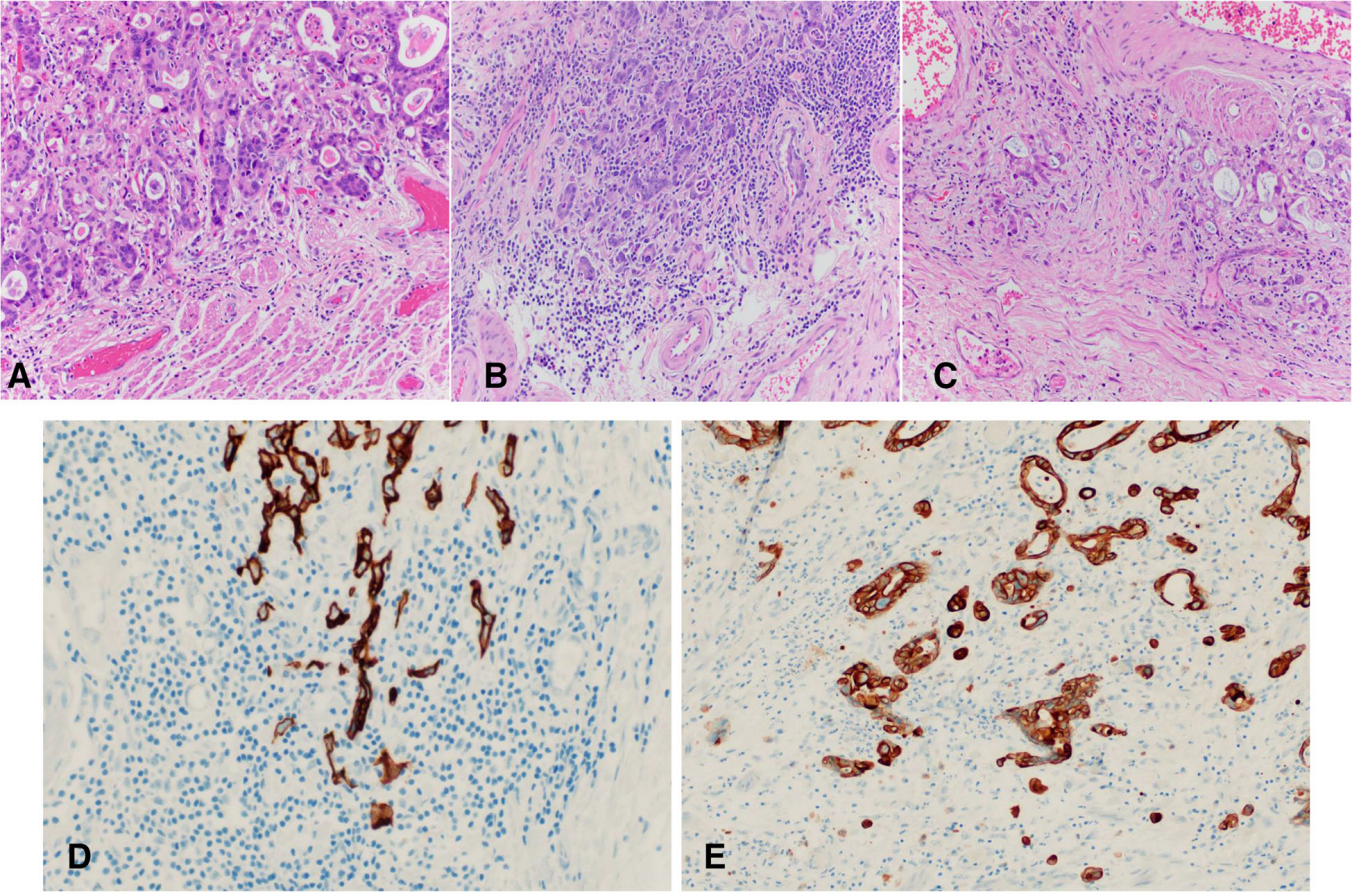
**A**. Low tumor budding. **B**. Intermediate tumor budding. Hematoxylin and Eosin stain × 400. **C**. High tumor budding. Hematoxylin and Eosin stain × 200. **D**. Pankeratin immunostain with intermediate tumor budding, × 400. **E**. Pankeratin immunostain with high tumor budding, × 400

DOI was classified using Vieth and Stolte [5] guidelines: m1, limited to the lamina propria mucosae; m2, involving the superficial muscularis mucosae (Fig. 3A); m3, involving the layer between the superficial and deep muscularis mucosae (Fig. 3B); and m4, involving the deep muscularis mucosae (Fig. 3C). Depth of submucosal invasion was categorized as sm1 (≤500 μm) (Fig. 3D); sm2 (> 500–1000 μm) (Fig. 3E); and sm3 (> 1000 μm) (Fig. 3F). Tumor budding was assessed at the advancing tumor edge (peritumoral) and scored as 1 (low), 2 (intermediate), or 3 (high) on hematoxylin and eosin-stained sections, using guidelines from the International Tumor Budding Consensus Conference for Colorectal Cancer [6] (Fig. 4A, B, C). Immunostain for pankeratin was performed in select cases to illustrate tumor budding for the manuscript (Fig. 4D and E). Peritumoral inflammation was categorized as mild or no significant peritumoral inflammation, lymphoplasmacytic with lymphoid aggregates, or neutrophil rich. Low-risk or high-risk features was defined according to ESGE guidelines [4]. Low-risk features included well to moderately differentiated adenocarcinoma with mucosal invasion (m1–m4) or sm1 invasion but no LVI. High-risk features included poor differentiation, LVI, or ≥ sm2 DOI.

**Fig. 5:**
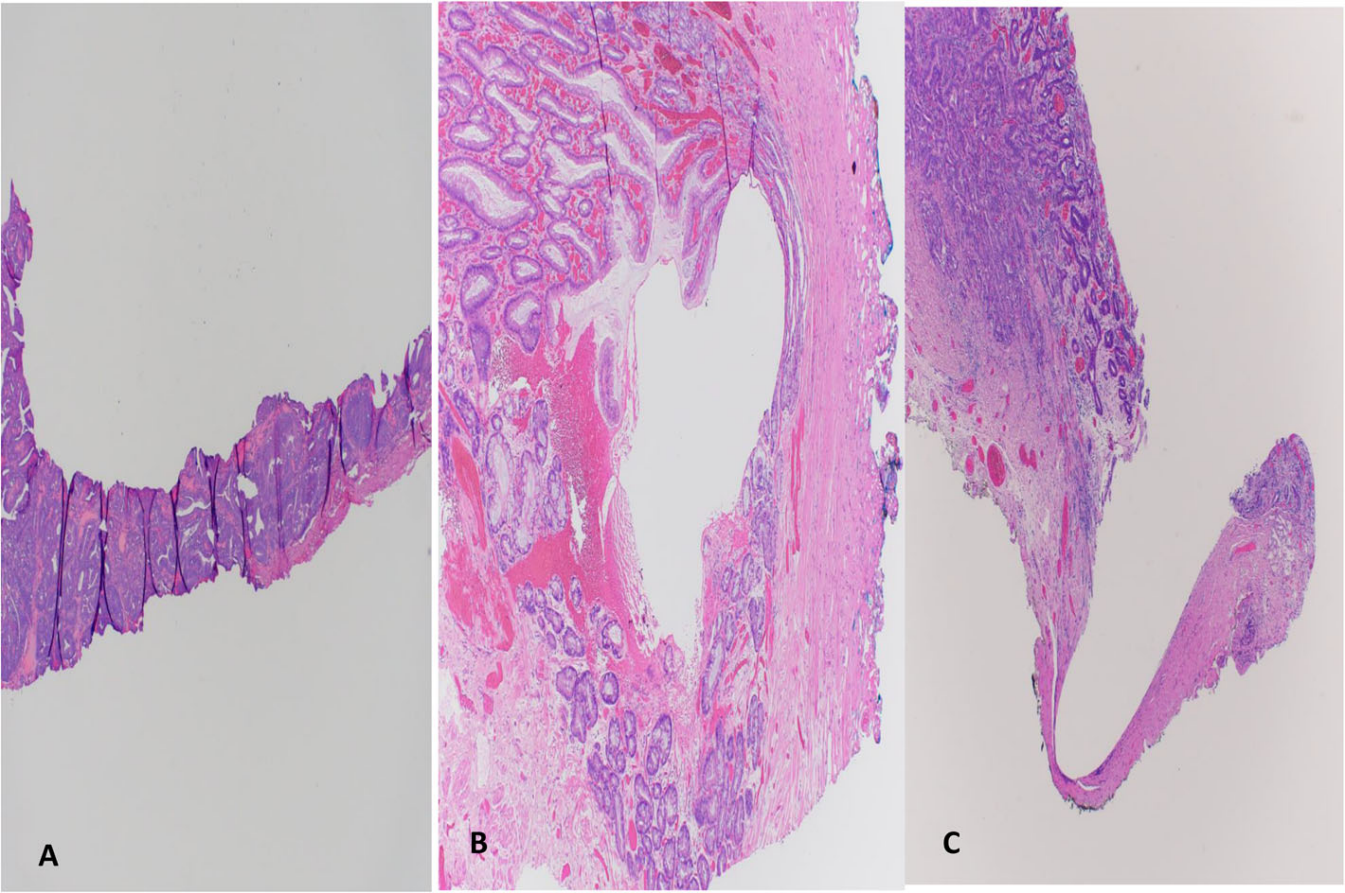
**A**. Tissue folding artefact due to improper processing and embedding. Hematoxylin and Eosin stain × 20. **B**. Pinhole artefact causing curling of tissue at edges leading to difficulty in peripheral margin interpretation. Hematoxylin and Eosin stain. × 40. **C**. Large pinhole artefact causing disruption of tissue at the edge. × 20

Specimen depth of excision was measured histologically using the Vieth and Stolte [5] criteria i.e. sm1 (≤500 μm); sm2 (> 500–1000 μm); and sm3 (> 1000 μm). Specimen processing quality was evaluated by assessing the presence of tissue folding (producing difficulty with interpreting DOI) (Fig. 5A), large pin artefacts at the tissue edge causing curling of tissue (Fig. 5B) or disruption of tissue at the margin (Fig. 5C), thereby, leading to difficulty in assessing margins.

## Results

The study group included 42 patients with ≥1 ESD resection. Three patients underwent 2 additional ESD resections and 1 patient underwent 1 additional ESD resection. Therefore, a total of 49 ESD cases/specimens from 42 patients were included in the study.

### Patient characteristics

Mean age of the 42 patients was 68 years (range: 46–83 years). There were 36 males (86%) and 6 females (14%). On final pathologic evaluation, 24 (57%) patients had intramucosal (T1a) EAC and 18 (43%) had submucosal (T1b) EAC.

### Detailed pathological descriptions of endoscopic submucosal dissection specimens

#### Invasive intramucosal adenocarcinoma (T1a EAC)

Twenty-four patients had T1a EAC: 23 males and 1 female. Eighteen (75%) T1a EAC patients had long-segment BE, while 6 (25%) had short-segment BE. Three patients underwent 2 repeat ESD resections. Findings in these repeat resections were as follows: intramucosal adenocarcinoma, 2 resections; high-grade dysplasia, 3 resections; and polypoid nondysplastic BE, 1 resection. A total of 26 T1a EAC were evaluated from 30 ESD resections in 24 patients.

##### *Gross evaluation (n = 30)* (Table 1)

ESD specimens were ranging between 2 to 12 cm in the longest dimension. The largest specimen measured 12 cm × 10 cm and was resected en bloc. Twenty-seven ESD resections were en bloc, while 3 were piecemeal resections. Specimen orientation was marked by the gastroenterologist in 14 specimens (47%). Gross evaluation revealed ≥1 tumor nodule in 20 specimens (67%); a unifocal dominant nodule was observed in 17 of these 20 resections (85%). Ulcerated mucosa was seen in 3 specimens (10%), and no definitive tumor was identified in 7 specimens (23%). Tumor nodularity size was < 3 cm in 12 (60%) of the 20 resections with grossly identifiable tumor and > 3 cm in the 8 (40%) of these resections. On gross evaluation, peripheral margins could be commented on 23 of 30 resections that showed gross lesions. The peripheral margin was negative in 19 (83%) resections and positive in 4 (17%) resections.

**Table 1:** Specimen Processing Data

Factor		Intramucosal Adenocarcinoma (***n*** = 30)^**a**^	Submucosal Adenocarcinoma (***n*** = 19)^**a**^
**Specimen size**	< 5 cm	18 (60%)	10 (52.7%)
	> 5 cm	12 (40%)	9 (47.4%)
**En bloc or Piecemeal**	En bloc	27 (90%)	18 (94.7%)
	2 pieces	1 (3.3%)	1 (5.3%)
	3 pieces	2 (6.7%)	0 (0%)
**Orientation marked by gastroenterologist**		14 (46.7%)	9 (47.4%)
**Gross examination findings**	Tumor nodule(s)	20 (66.7%)*	17 (89.5%)**
	Ulcerated mucosa	3 (10%)	0 (0%)
	No tumor	7 (23.3%)	2 (10.5%)
**Peripheral margin involvement (by histology)**	Negative	25 (83.3%)	10 (52.6%)
	Positive	3 (10%)	2 (10.5%)
**Changes in peripheral margin involvement from gross to histologic evaluation**	Positive to negative	1 (3.3%)	0 (0%)
	Negative to positive	1 (3.3%)	7 (36.8%)
	Sm1 (< 500 μ)	7 (23%)	4 (21%)
**Specimen depth of excision (microscopic)**	Sm2 (> 500-1000 μ)	21 (70%)	12 (63%)
	Sm3 (> 1000 μ)	2 (7%)	3 (16%)
**Large pinholes**		2 (6.7%)	6 (31.6%)
**Tissue folding, producing interpretation problems**		3 (10%)	2 (10.5%)

Statistics presented as Frequency (%)

a Data are from 30 endoscopic submucosal dissection (ESD) resections in 24 patients, of which 26 were positive for intramucosal adenocarcinoma

b Data are from 19 ESD resections in 18 patients, of which 18 were positive for submucosal adenocarcinoma

*Multinodularity in 3 specimens

**Multinodularity in 5 specimens

##### *Histologic evaluation* (Table 2)

Intestinal metaplasia in nondysplastic columnar mucosa was seen in all 30 resections. Duplication of the muscularis mucosae was also observed in all resections. The specimen depth of excision in the 30 resections was as follows: sm1 *n* = 7 (23%), sm2 *n* = 13 (70%) and sm3 *n* = 2 (7%) (Table 1). The Vieth and Stolte tumor depth of invasion (DOI) in the 26 T1a EAC resections was as follows: m1, 0 tumors; m2, 7 tumors (27%); m3, 16 tumors (61%); and m4, 3 tumors (12%). Tumors were well differentiated in 18 cases (69%), moderately differentiated in 7 cases (27%), and 1 case (4%) showed focal poor differentiation.

**Table 2:** Histologic features of intramucosal and submucosal adenocarcinoma

Feature		Intramucosal adenocarcinoma (***n*** = 26)^**a**^	Submucosal adenocarcinoma (***n*** = 18)^**b**^
Histologic type	Tubuloinfiltrative	13 (50%)	8 (44.4%)
	Tubulocystic	9 (34.6%)	4 (22.2%)
	Mixed	3 (11.5%)	1 (5.5%)
	Papillary	1 (3.8%)	2 (11.1%)
	Mixed tubular and Mucinous	0 (0%)	2 (11.1%)
	Mixed tubular and signet ring cell	0 (0%)	1 (5.5%)
	Pure mucinous	0 (0%)	0 (0%)
	Pure signet ring cell	0 (0%)	0 (0%)
Tumor differentiation	Well differentiated	18 (69.2%)	4 (22.2%)
	Moderately differentiated	7 (26.9%)	7 (38.9%)
	Poorly differentiated	1 (3.8%)	7 (38.9%)
Lymphovascular invasion		0 (0%)	4 (22.2%)
Large vessel invasion		0 (0%)	1 (5.5%)
Perineural invasion		0 (0%)	1 (5.5%)
Tumor budding	Low	0 (0%)	1 (5.5%)
	Intermediate or high	0 (0%)	7 (38.9%)
Peritumoral inflammatory response	Lymphoplasmacytic inflammation with lymphoid aggregates	6 (23%)	7 (38.9%)
	Neutrophilic inflammation	0 (0%)	2 (11.1%)
Desmoplasia		0 (0%)	8 (44.4%)
Depth of invasion		m1 = 0 (0%) m2 = 7 (26.9%) m3 = 16 (61.5%) m4 = 3 (11.5%)	sm1 = 12 (66.7%) sm2 = 3 (16.7%) >sm2 = 3 (16.7%)

Statistics presented as Frequency (%)

a: Data are from 26 endoscopic submucosal dissection (ESD) resection specimens positive for intramucosal adenocarcinoma

b: Data are from 18 ESD resection specimens positive for submucosal adenocarcinoma

Tumor histology pattern was tubular in 25 cases (96%) and papillary in 1 case (4%). No mucinous or signet ring cell patterns were seen. Tubular patterns were tubuloinfiltrative in 13 cases (50%), tubulocystic in 9 cases (35%), mixed tubuloinfiltrative and tubulocystic in 3 cases (11%) and papillary in 1 case (4%). No LVI, large-vessel invasion, perineural invasion, or tumor budding were observed. Desmoplasia was not seen, although 1 case exhibited myxoid stromal change. Peritumoral inflammation characterized by lymphoplasmacytic inflammation with lymphoid aggregate was seen in 6 (23%) of the 26 cases. No peritumoral neutrophilic inflammation was observed. Folding of tissue sections and difficulties with DOI assessment, was encountered in 3 (10%) of the 30 resections. These folds were corrected by melting the paraffin block and re-embedding the tissue. A large pinhole artefact leading to curling or disruption of tissue edges was seen in 2 (7%) of the 30 resections (Fig. 5B and C).

##### Margin status and outcomes (Tables 3, 4 and 5)

Of the 26 ESD resections with intramucosal adenocarcinoma, 19 (73%) were curative resections, with both mucosal and deep margins negative for invasive adenocarcinoma. The remaining 7 (27%) T1a EAC resections were noncurative resections of which 6 were R1 resections (with positive margins) and one showed tumor with focal poor differentiation. Of R1 resections, 5 had a peripheral mucosal margin positive for adenocarcinoma, with a concurrent positive deep margin in 2 patients. RFA was performed 3 months later in all 6 patients. Two patients with only peripheral margin positivity underwent 2 repeat ESD procedures, followed by eventual R0 resection. Both of these patients have been negative for dysplasia or carcinoma on follow-up endoscopies for 9 and 14 months. The other patient with only a positive peripheral margin underwent minimally invasive distal esophagectomy because of high suspicion of extensive multifocal disease according to endoscopic ultrasound (EUS) evaluation (Table 3). The esophagectomy pathology was negative for residual invasive adenocarcinoma and showed BE with low- and high-grade dysplasia.

**Table 3:** Patients who underwent esophagectomy (*n* = 11)

Adenocarcinoma Stage	Degree of tumor differentiation	Depth of invasion	LVI	Deep margin	Peripheral margin	Esophagectomy pathology AJCC/CAP staging
Intramucosal	Well	m3	No	Negative	Positive	pT0N0
Intramucosal	Poor	m3	No	Negative	Negative	pT1aN0
Intramucosal	Well	m3	No	Positive	Negative	pT1aN0
Submucosal	Poor	sm2	Yes	Negative	Negative	pT1bN0
Submucosal	Poor	sm1	Yes	Negative	Negative	pT1aN1
Submucosal	Moderate	>sm2	No	Positive	Positive	pT1bN0
Submucosal	Moderate	>sm1	No	Positive	Positive	pT2N0
Submucosal	Moderate	sm2	No	Positive	Negative	pT0N0
Submucosal	Well	sm1	No	Negative	Positive	pT0N0
Submucosal	Poor	sm2	No	Positive*	Positive	pT1aN0
Submucosal	Poor	sm1	No	Negative	Positive	pT2N0

*AJCC/CAP* American Joint Commission on Cancer/College of American Pathologists; *LVI* lymphovascular invasion;

m3 involving the layer between the superficial and deep muscularis mucosae; sm1 submucosal invasion ≤500 μm, sm2 submucosal invasion > 500 μm,

* Single atypical gland in cauterized tissue at margin

**Table 4:** Patients with positive deep margins (*n* = 10)

Adenocarcinoma Stage		Tumor morphology	Deep margin	Follow-up
Intramucosal		Low-risk features	Positive at site of tissue disruption with cautery effect	6 month follow-up endoscopy and biopsies negative for carcinoma
Intramucosal		Low-risk features	Positive at the site of tissue disruption with cautery artifact	11 month follow-up with no recurrent carcinoma, just BE with low-grade dysplasia treated with RFA
Intramucosal*		Low-risk features	Plane of resection “mucosal” at the site of positive deep margin	Esophagectomy showed residual tumor, pT1aN0 (AJCC/CAP staging, 8th edition)
Submucosal		High-risk features *(DOI: sm2)*	Tumor present at the edge of resection with both peripheral and deep margin positive	Esophagectomy showed residual tumor, pT1bN0 (AJCC/CAP staging, 8th edition)
Submucosal		Low-risk features	Plane of resection “mucosal” at the site of positive deep margin	3 and 9-month endoscopy with biopsy of ESD scar site showed BE but no dysplasia or carcinoma
Submucosal		Low-risk features *(High-grade tumor budding)*	Tumor present at the edge of resection	Esophagectomy showed residual tumor, pT2N0 (AJCC/CAP staging, 8th edition)
Submucosal		High-risk features *(DOI: sm2)*	Plane of resection “mucosal” at the site of positive deep margin	Esophagectomy showed no residual tumor, pT0N0 (AJCC/CAP staging, 8th edition)
Submucosal**		High-risk features *(Poorly differentiated tumor & Large-vessel invasion)*	Positive deep margin	Referred for more chemoradiation
Submucosal**		High-risk features *(Poorly differentiated* *Tumor, LVI present)*	Positive deep margin	Referred for more chemoradiation
Submucosal		High-risk features *(DOI: 980/1500 μm, sm2)*	Single atypical gland in cauterized tissue at the deep margin	Esophagectomy showed residual tumor; pT1aN0 (AJCC/CAP staging, 8th edition)

*AJCC/CAP* American Joint Commission on Cancer/College of American Pathologists; *BE* Barrett’s esophagus; *DOI* depth of invasion; *ESD* endoscopic submucosal dissection; *LVI* lymphovascular invasion; *RFA* radiofrequency ablation

* Patient had esophageal stricture resistant to endoscopic intervention

**ESD was a debulking procedure post-chemoradiation in a patient with known esophageal adenocarcinoma

**Table 5:** Patient follow up

Adenocarcinoma (number of patients)	Resection risk profile	Follow up
Intramucosal adenocarcinoma, EAC T1a (*n* = 24)		
	R0 resection and low risk features (*n* = 17)	Endoscopic surveillance
	R0 resection and high risk features (*n* = 1)	Endoscopic surveillance for 27 months, developed recurrence and underwent esophagectomy
	R1 resection and low risk features (*n* = 6)	2 patients followed by endoscopic surveillance
		2 patients underwent esophagectomy
		2 patients underwent ESD ×2
Submucosal adenocarcinoma EAC T1b (*n* = 18)	R0 resection and low risk features (*n* = 3)	Endoscopic surveillance
	R0 resection and high risk features (*n* = 4)	1 patient followed by endoscopic surveillance
		2 patients underwent esophagectomy
		1 patient developed liver metastasis
	R1 resection and low risk features (*n* = 5)	4 patients followed by endoscopic surveillance
		1 patient underwent esophagectomy
	R1 resection and high risk features (*n* = 6)	4 patients underwent esophagectomy
		2 patients got chemoradiation (ESD was a debulking procedure)

Three patients had positive deep margins, 2 of who had concurrent peripheral margin positivity (Table 4). In one patient with both positive deep and peripheral margins, the tumor was at the edge of resection and “mucosal” at the site of positive margins. This patient had severe fibrosis at the time of ESD with an esophageal stricture resistant to therapeutic intervention. Subsequent esophagectomy showed a 0.7 cm focus of residual intramucosal adenocarcinoma and no lymph node metastasis (American Joint Commission on Cancer/College of American Pathologists [AJCC/CAP] pathologic stage pT1aN0). In other two patients with positive deep margins, the specimens showed tissue disruption with cautery artefact at the site of the positive deep margin (Fig. 5). They underwent follow-up with endoscopy and biopsies for 9 to 11 months. During this time, no invasive adenocarcinoma was observed, although recurrent low-grade dysplasia was observed in 1 patient and was treated with RFA.

The only T1a EAC patient with high risk features (focal poor differentiation) had an R0 resection and was negative for dysplasia or recurrent carcinoma on follow-up endoscopy for 19 months. He then presented with recurrent adenocarcinoma with signet ring cells in biopsy at 27 months follow up endoscopy. He underwent esophagectomy that showed an intramucosal signet ring cell carcinoma and no lymph node metastasis (AJCC/CAP pathologic stage pT1aN0).

Overall, 24 (92%) of the 26 intramucosal adenocarcinomas had good outcomes or accumulatively curative ESD resections with RFA ablation**.**

#### Invasive submucosal adenocarcinoma (T1b EAC)

Eighteen patients had T1b EAC: 13 males and 5 females. Twelve patients (67%) had long-segment BE and 6 (33%) had short-segment BE. One patient underwent 1 repeat ESD resection, which was negative for residual carcinoma or dysplasia. A total of 18 T1b EAC resections were evaluated from 19 ESD resection procedures in 18 patients.

##### *Gross evaluation (n = 19)* (Table 1)

ESD resection specimens were ranging between 2 and 10 cm in the longest dimension. All except 1 resection were en bloc (95%). The largest ESD resection specimen, which was an en bloc resection, was 8 cm by 10 cm. The sole piecemeal resection consisted of 2 pieces. Specimen orientation was marked in 9 resections (47%). Gross evaluation showed ≥1 tumor nodule in 17 (89%) of the 19 resections: a unifocal dominant nodule was observed in 12, while 5 resections exhibited multinodularity. No definitive tumor was identified on gross examination in 2 resections (11%). The dominant tumor size was < 3 cm in 5 resections and > 3 cm in 12 resections. By gross examination, peripheral margins could be commented on 17 of 19 specimens that showed gross lesions. The peripheral margins were negative in 15 resections and positive in 2 resections.

##### *Histologic evaluation* (Table 2)

Intestinal metaplasia in nondysplastic columnar mucosa was present in all 19 resection specimens. Duplication of the muscularis mucosae was also observed in all resection specimens. The specimen depth of excision in the 19 resections was as follows: sm1 *n* = 4 (21%), sm2 *n* = 12 (63%) and sm3 *n* = 3 (16%) (Table 1). The Vieth and Stolte DOI for the 18 T1b EAC specimens was as follows: sm1, 12 tumors (67%); sm2, 3 tumors (16.5%); and > sm2, 3 tumors (16.5%), all of which had a positive deep margin (Table 4). A component of poor differentiation (histologic grade 3) was noted in 7 tumors (39%), and well to moderately differentiated morphology (grades 1 or 2) observed in the remaining 11 tumors (61%). Tumor histology pattern was tubular in 13 cases (72%), papillary in 2 cases (11%), mucinous differentiation involving 40% of tubular adenocarcinoma was seen in 2 cases (11%), and signet ring cell component comprising 30% of tubular adenocarcinoma was seen in 1 case (6%). No case of pure mucinous adenocarcinoma or pure signet ring cell carcinoma was seen. The tubular patterns were tubuloinfiltrative in 8 cases (61%), tubulocystic in 4 cases (31%), and mixed tubuloinfiltrative and tubulocystic in 1 case (8%). LVI was present in 4 cases (22%), whereas large-vessel invasion was observed in 1 case (5.5%) and perineural invasion in 1 case (5.5%). Tumor budding was present at the advancing edge of the tumor in 8 cases (39%); budding was rated as low-grade in 1 case (14%) and intermediate- or high-grade in 7 cases (86%) (Table 6). Peritumoral inflammation was observed in 9 tumors (50%), consisting of significant peritumoral lymphoplasmacytic inflammation with lymphoid aggregates in 7 cases and peritumoral neutrophilic inflammation in 2 cases. No significant peritumoral inflammation was seen in the remaining 9 tumors (50%). Desmoplasia was observed in 8 tumors (44%). Folding of tissue sections producing difficulties during DOI assessment was present in 2 (11%) of 19 submucosal resection specimens, but it was corrected by melting the paraffin block and re-embedding the tissue. A large pinhole artefact leading to curling of tissue edges occurred in 6 resection specimens (33%)(Fig. 5B and C).

**Table 6:** Tumor budding and outcomes (*n* = 8)

Risk stratification based on morphology	Tumor budding score	Outcome
*High-risk features* Poorly differentiated Perineural invasion DOI: sm3	1	Esophagectomy with residual tumor, pT1bN0
*High-risk features* Poorly differentiated LVI present DOI: sm1	3	Esophagectomy with residual tumor, pT1aN1
*High-risk features* Poorly differentiated DOI: sm1	2	Liver metastases
*High-risk features* DOI: sm2 R1 resection, deep and peripheral margin positive	3	Esophagectomy with residual tumor, pT1bN0
*Low-risk morphology* R1 resection, deep and peripheral margin positive	3	Esophagectomy with residual tumor, pT2N0
High-risk features* Poorly differentiated morphology Large-vessel invasion	2	Known case of esophageal adenocarcinoma, not surgical candidate, prior history of neoadjuvant chemotherapy, ESD performed for debulking
High-risk features* Poorly differentiated adenocarcinoma LVI present	2	Known case of esophageal adenocarcinoma, not surgical candidate, prior history of neoadjuvant chemotherapy, ESD performed for debulking
*High-risk features* Poorly differentiated adenocarcinoma Deep margin negative Peripheral margin positive	3	Esophagectomy: residual adenocarcinoma pT2N0

*DOI*, depth of invasion; *ESD* endoscopic submucosal dissection; *LVI* lymphovascular invasion; *p* pathologic

##### Margin status and outcomes (Tables 3, 4 and 5)

Seven T1b EAC patients had R0 resection (39%). Of these 7 tumors, 3 had low-risk features and 4 exhibited high-risk features. All three patients with low-risk features and R0 resection had no evidence of dysplasia, recurrent carcinoma, or metastasis during 7 to 11 months follow-up. Two of the 4 patients with high-risk features and R0 resection underwent esophagectomy during follow-up (Table 3). At esophagectomy, 1 patient had node-positive residual intramucosal adenocarcinoma (American Joint Commission on Cancer/College of American Pathologists [AJCC/CAP] pathologic stage pT1aN1), whereas the other patient had node-negative submucosal adenocarcinoma (AJCC/CAP stage, pT1bN0). The latter patient had a focus of perineural invasion at ESD resection. The third patient with high-risk features developed liver metastasis. The fourth patient with high-risk features refused esophagectomy, and subsequent endoscopies during 11 months of follow-up have been negative.

**Fig. 6:**
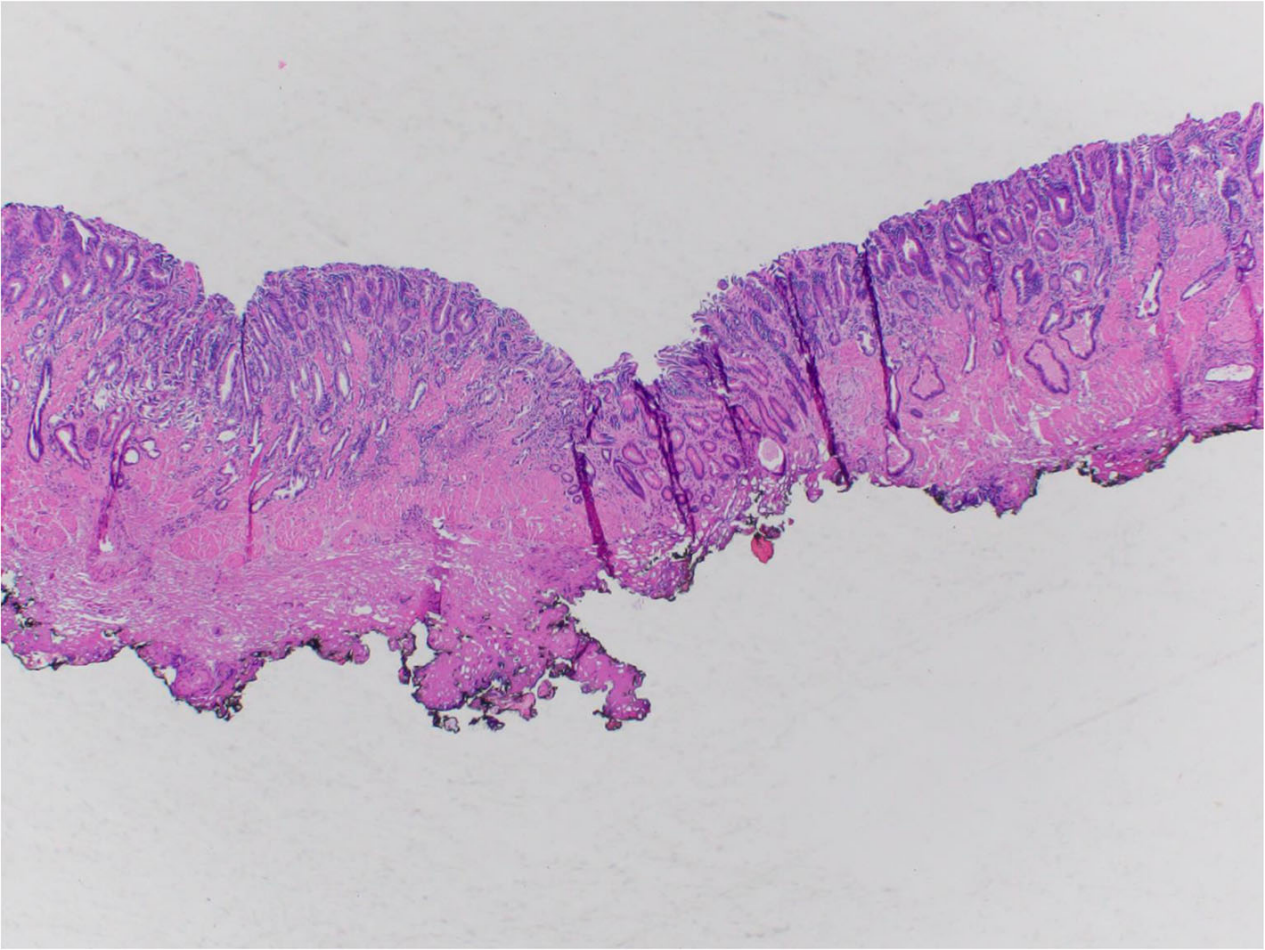
Deep margin, positive for tumor due to superficial plane of resection. Hematoxylin and Eosin stain. × 40

Eleven T1b EAC patients had R1 resections (61%): 5 had low-risk features and 6 had high-risk features. All 5 tumors with low-risk features and R1 resection had positive peripheral margins, 2 of which also had positive deep margins for adenocarcinoma (Table 4). One patient with low-risk features and a positive deep margin had a superficial (mucosal) plane of resection at the site of the positive margin, in contrast to a submucosal plane of resection in other parts of the specimen (Fig. 6). Follow-up endoscopy and biopsy of the scar site 4 months later showed residual BE but no dysplasia or adenocarcinoma. The second patient with low-risk features and a positive deep margin underwent esophagectomy, which revealed node-negative residual adenocarcinoma invading the muscularis propria (AJCC/CAP stage, pT2N0) (Table 3). All 3 patients with low-risk features and only positive peripheral margins had nondysplastic BE on follow-up endoscopies.

Of the 6 submucosal adenocarcinomas with high-risk features and R1 resection, deep margins were positive in 5 cases (Table 4), peripheral margins were positive in 4 cases, and both deep and peripheral margins were positive in 3 cases. Of the cases with positive deep margins, 1 submucosal adenocarcinoma (sm2 DOI) had a deep margin positive for intramucosal adenocarcinoma because the resection plane was “mucosal” at the site of the positive margin. At follow-up esophagectomy, no residual tumor was observed in this patient (AJCC/CAP stage, pT0N0) (Table 3). In another submucosal adenocarcinoma (sm2), the deep margin was positive for a single atypical gland in the cauterized submucosal tissue, and follow-up esophagectomy revealed node-negative residual intramucosal adenocarcinoma (AJCC/CAP stage, pT1aN0). Another patient with submucosal adenocarcinoma (sm2) and a positive deep margin underwent esophagectomy, which showed node-negative residual submucosal adenocarcinoma (AJCC/CAP stage, pT1bN0). The fourth and fifth cases of submucosal adenocarcinoma with high-risk features and positive deep margins were patients with known BE-related adenocarcinoma treated with chemoradiation; ESD resection was performed as a debulking procedure because the patients were poor surgical candidates. The patient with high-risk features and a positive peripheral margin but negative deep margin underwent esophagectomy, which revealed node-negative residual adenocarcinoma invading the muscularis propria (AJCC/CAP stage, pT2N0).

#### Associations between histologic features and outcomes (Table 7)

Poor tumor differentiation, submucosal DOI > 500 μm (≥sm2), lymphovascular invasion, tumor budding and tubuloinfiltrative morphology (versus tubulocystic morphology) were frequently associated with poor outcomes. Tumor budding when present was associated with other high-risk features.

**Table 7:** Pathologic variables associated with poor outcomes and better outcomes in ESD resections

Poor outcomes*	Poor tumor differentiation
	Submucosal depth of invasion > 500 μm (> sm2)
	Lymphovascular invasion
	Large vessel invasion
	Positive margins, specifically, positive deep margin
Better outcomes*	Well to moderately differentiated tumor morphology
	Submucosal depth of invasion < 500 μm (sm1)
	Negative margins, specifically, negative deep margin
	Absence of lymphovascular invasion

* Our study also observed a trend in poor outcomes with tubulo-infiltrative morphology (versus tubulocystic morphology) and tumor budding. However, these could not be confirmed as independent risk factors because they were seen in association with other high risk histologic features

## Discussion

ESD is a relatively new technique in the United States for managing early esophageal adenocarcinoma. It is a labor-intensive procedure requiring high-level expertise from gastroenterologists, as well as pathologists, and is currently performed at tertiary care centers by highly skilled interventional gastroenterologists. Most data and recommendations regarding ESD in BE-related adenocarcinoma have originated from the Asian literature, with few studies from the United States. ESGE published guidelines about the role of ESD in BE-associated adenocarcinoma and provided recommendations for additional therapeutic management based on the histopathology results of ESD resections [4]. The recommendations regarding further management included the following: 1) endoscopic en bloc R0 resection of mucosal adenocarcinoma is curative; 2) endoscopic en bloc R0 resection of sm1 lesions (< 500 μm) with a low-risk profile (well to moderately differentiated adenocarcinoma and no LVI) is potentially curative, and in multidisciplinary discussion, the risks of surgery should be balanced against the risk of lymph node metastasis; 3) surgery is recommended in the presence of LVI, a poorly differentiated tumor, DOI deeper than sm1 (> 500 μm), or positive vertical margins; 4) endoscopic surveillance/retreatment is recommended over surgery when the horizontal margin is positive or resection is piecemeal, if no other high-risk criteria are present; and 5) further treatments are necessary (eg, EMR, RFA) after curative resection in patients with early neoplasia in BE to ablate or remove residual metaplastic epithelium where foci of synchronous intraepithelial neoplasia could be overlooked and metachronous lesions could arise.

ESGE’s recommendation for additional treatment after ESD resection of adenocarcinoma exhibiting high-risk features, such as poor tumor differentiation, LVI, or submucosal DOI > 500 μm, is based on the results of multiple studies reporting a high risk of lymph node metastasis in these patients. These studies were performed predominantly on esophagectomy specimens [7–11]; only a few studies used EMR specimens [12–14]. Two studies [15, 16] have involved patients undergoing ESD in Japan, but no published study has examined patients undergoing ESD in the United States.

Ours is a descriptive study where we report our results of 49 ESD resections in 42 patients with BE-associated adenocarcinoma managed according to ESGE guidelines, with multidisciplinary discussions and referring patients for additional therapy when appropriate based on ESD pathology. Esophagectomy was performed in 11 patients: 8 with submucosal adenocarcinoma and 3 with intramucosal adenocarcinoma detected in ESD resection specimens. Nine of these patients were referred for esophagectomy because ≥1 high-risk feature and/or positive deep margin was observed on ESD resection, in accordance with ESGE recommendations. Residual adenocarcinoma was detected in esophagectomy specimens from 8 of these 9 patients, 1 of who had lymph node metastasis. We found poor outcomes in cases that showed high risk features such as poorly differentiated tumors, submucosal DOI > 500 μm, or LVI. Our rate of lymph node metastasis (11.1%) was comparable to rates (10–19.9%) reported previously for superficial BE-related adenocarcinoma [7–14]. In a study of ESD resection of 87 gastric cardia adenocarcinomas and 55 BE-associated adenocarcinomas, Osumi et al. found no lymph node metastasis in 70% of patients who underwent additional surgery [16]. High-risk features in endoscopic resection specimens of BE-related superficial adenocarcinoma adversely affect survival and recurrence rates, which are similar whether node-positive or node-negative residual pT1 adenocarcinoma is found on subsequent esophagectomy [11]. Overall, our findings provide further evidence supporting ESGE recommendations of additional therapy in patients with high-risk pathologic features.

In addition to the high-risk features described above, we noted tumor budding in 44% of patients with submucosal adenocarcinoma. No peritumoral tumor budding was observed in patients with intramucosal adenocarcinoma. The budding was intermediate- to high-grade in 7 of the 8 patients with tumor budding. All tumors with peritumoral tumor budding also exhibited ≥1 other high-risk features and were therefore triaged as per ESGE guidelines. Tumor budding was more frequently associated with tubular adenocarcinomas with a component of mucinous or signet ring cell patterns than with pure tubular adenocarcinomas. Imai first described tumor budding in 1954 as “sprouting” at the invasive edge of carcinoma [17], and revised definitions have appeared over the years. A recent consensus conference on colorectal carcinoma defined tumor budding (based on routine hematoxylin and eosin staining) as the presence of 1 tumor cell or a cluster of < 5 tumor cells in a hotspot (area of maximal budding) at 200× magnification and categorized budding as low- (0–4 buds), intermediate- (5–9 buds), or high-grade (> 10 buds) [6].

Tumor budding is biologically associated with down-regulation of E-cadherin expression and nuclear translocation of beta-catenin, leading to activation of WNT signaling and resulting in epithelial to mesenchymal transformation, facilitating metastasis [18]. Tumor budding has been reported in several studies as a strong independent predictor for metastasis and aggressive phenotype in colorectal, pancreatic, gastric, and esophageal squamous cell carcinoma [19]. The few studies evaluating tumor budding in esophageal adenocarcinoma reported that it had poor prognostic value and was an independent risk factor for lymph node metastasis and associated with aggressive tumor phenotype [20–22].

Esophageal adenocarcinomas with a tubular pattern had 2 distinct morphologies: tubuloinfiltrative or tubulocystic. Although these morphologic patterns have been mentioned briefly in the literature [23, 24], their clinical significance has not been widely researched. Only one previous study investigated the relevance of these patterns for risk of metastasis [23]. In that retrospective study of 357 patients, the tubuloinfiltrative pattern was significantly associated with metastasis in univariate analysis. We noted frequent poor outcomes in cases with tubuloinfiltrative pattern.

Margin involvement by tumor at endoscopic resection is associated with tumor recurrence. Studies of EMR for early gastroesophageal cancers reported recurrence risks of 37 to 50% in the presence of positive margins [12, 25, 26]. In a series of EMR for BE-related neoplasia, the peripheral margin was positive in 68% of tumors, and both peripheral and deep margins were positive in 28% of cases [27]. As per ESGE guidelines, positive vertical or deep margins warrant additional treatment. Deep margins were positive in 10 ESD resections (3 intramucosal and 7 submucosal adenocarcinomas). In 4 of these cases (2 intramucosal and 2 submucosal), the positive deep margin was located in a focus of tissue disruption with associated cautery artefact. This focus was distant from the site of deepest invasion by tumor and was positive for tumor. In 3 of these cases, the positive margin was attributed to technical difficulty in ESD resection due to underlying fibrosis and esophagectomy was not performed after multidisciplinary discussion and discussion with patient. No recurrences have been observed during 6 to 11 months of endoscopic follow-up. In the 4th case, the esophagectomy was performed because of the presence of other high-risk features, but no residual tumor or lymph node metastasis was found. In another case, the deep margin was positive because a single atypical gland was observed in the cauterized tissue at the site of deepest invasion by tumor. Esophagectomy in that patient revealed a node-negative residual pT1a tumor. These findings indicate that deep margin can be positive due to technical difficulties in endoscopic resection. Any morphologic oddities related to positive deep margins be commented upon in the pathology report and discussed with the gastroenterologist to facilitate development of an appropriate management strategy.

Two patients underwent esophagectomy for high suspicion of extensive mucosal adenocarcinoma based on EUS evaluation. Both patients (1 T1a EAC and 1 T1b EAC) had low-risk tumor morphology but positive peripheral margins for intramucosal adenocarcinoma on ESD resections. Based on ESGE guidelines, these patients should have been managed with endoscopic surveillance and treatment. Esophagectomy specimens of both patients were negative for residual adenocarcinoma, and 1 of these patients died from surgical complications. These 2 cases highlight the limitations of EUS evaluation when assessing the extent and depth of BE-associated neoplasia, as has been previously reported [28–30]. EUS evaluation tends to overstage or understage BE-associated neoplasia, likely because of duplication of the muscularis mucosae, which is a phenomenon unique to BE. Therefore, EUS has very limited value in determining appropriate patient selection for endoscopic resection or esophagectomy. When available, advanced endoscopy techniques, such as narrow band imaging with magnified endoscopy or volumetric laser endomicroscopy using infra-red light, may be more helpful [30].

**Fig. 7:**
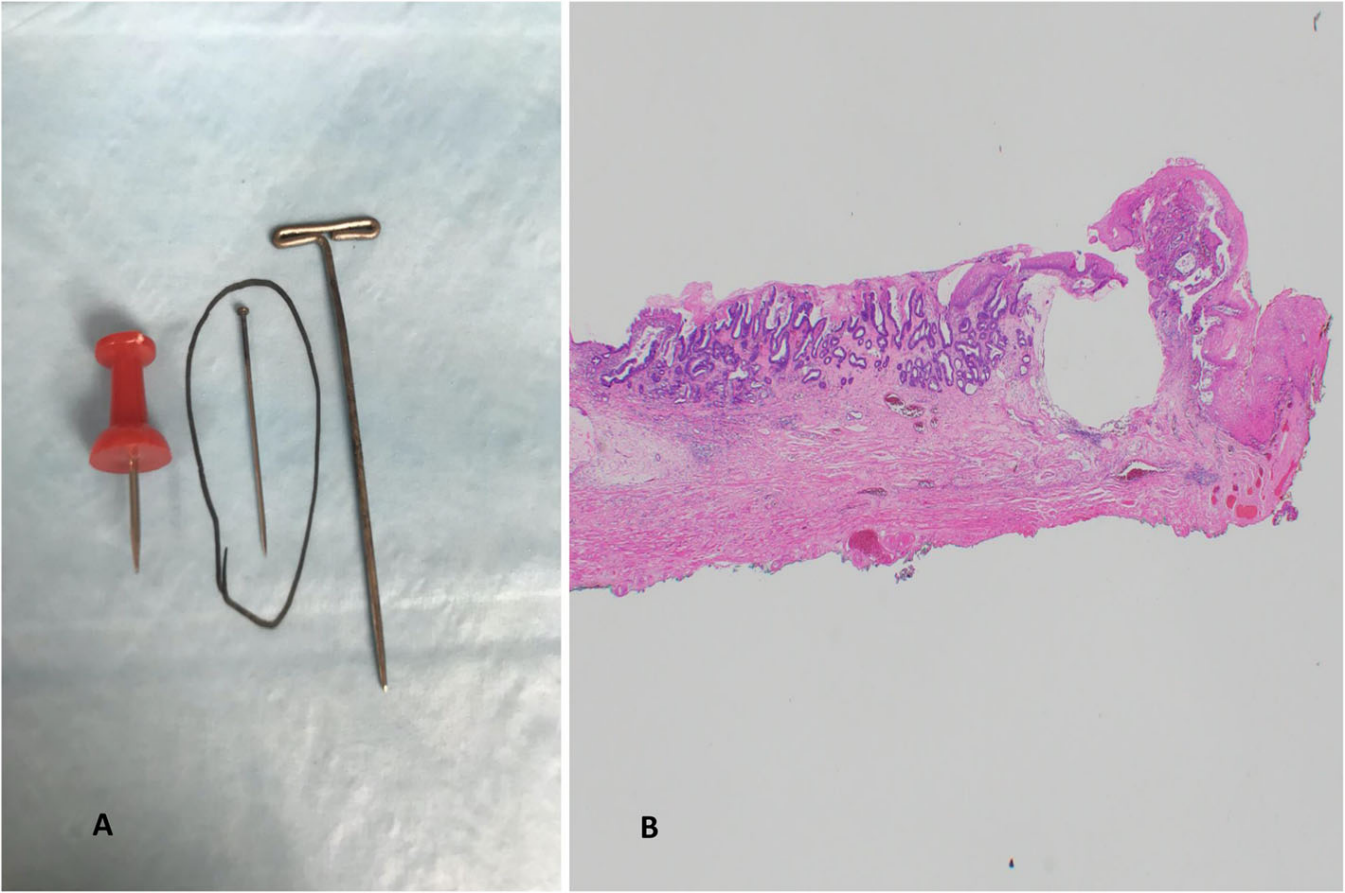
**A**. Recommended thin paper pins for pinning the tissue on the board for proper fixation. **B**. Small pin hole and excellent orientation of the tissue edges for optimal assessment of peripheral margins. Hematoxylin and Eosin stain × 20

Pathologic handling and processing of ESD resection specimens were performed in accordance with recommended guidelines to achieve optimal orientation of tissue sections for accurate assessment of DOI, margins, and other histological features that influence the need for additional treatment [2, 3]. In our experience, certain details are helpful for achieving optimal sections: 1) education and supervision of the technical staff involved in embedding; 2) tissue strips 1.5 to 2 cm in length (instead of shorter or longer strips), were easy to embed on edge and yielded well-oriented sections; 3) use of foam in cassettes to hold the tissue straight and help avoid tissue folding when tissue strips are > 2 cm in length; and 4) use of thin paper pins (rather than T-pins or push pins), which lead to better tissue preservation at the margins and smaller pinhole artefacts (Fig. 7A, B). On gross evaluation, tumor nodularity was more readily appreciable in submucosal tumors than in intramucosal adenocarcinomas. Histological assessment of Vieth and Stolte tumor DOI was performed with relative ease in ESD resection specimens because of the availability of long sections with intact tissue, which facilitated determination of histologic landmarks.

The strengths of our study include the use of good quality sections from ESD resection specimens because of direct supervision and education of pathology assistants and technical staff involved in the handling and grossing of the specimens, detailed histologic assessment by a single gastrointestinal pathologist, and access to pathology reports of esophagectomy specimens in a number of cases for follow-up. Limitations of the study include its single center design, with a relatively small sample size and limited duration of follow-up. As ESD resection is a relatively new technique in the United States, it will require a few more years to acquire a large series of patients with long term follow up. Some novel findings in this study include: tumors with tubuloinfiltrative pattern observed more frequently in cases with poor outcomes; deep margin could be falsely positive due to technical issues during resection. We noted tumor budding in association with other high-risk features and poor outcomes. This is a relatively less explored topic in BE-related adenocarcinoma. We describe our experience and issues with processing and handling of tissues, with suggestions to get optimal sections.

In conclusion, ESD specimens provide a unique opportunity to accurately assess the presence of curative resection and determine the need for additional therapy in superficial BE-related adenocarcinomas. Our study showed that curative ESD resection with RFA ablation was achieved in 92% of patients with intramucosal adenocarcinoma. Good outcomes were also noted in 50% of submucosal adenocarcinomas. Our results confirm the association between high-risk features (poor tumor differentiation, LVI, and submucosal DOI > 500 μm) in superficial esophageal adenocarcinoma and poor outcomes, indicating the need for additional treatment in these cases and validating current ESGE recommendations. We also noted tumor budding in esophageal adenocarcinoma and observed it be often present in association with other high-risk features. Of the 2 tubular histologic patterns of esophageal adenocarcinoma, the tubuloinfiltrative variant was more frequently associated with poorer outcomes. While ESGE guidelines recommend additional therapy when deep margins are positive, we found that this may not always be necessary, as a positive deep margin could be secondary to technical difficulties in performing ESD because of underlying fibrosis. When a positive deep margin is distant from the focus of deepest tumor invasion, a close endoscopic surveillance with biopsies may be a consideration.

## Data Availability

After IRB approval the data was collected from patient’s EPIC records by the PI (MOO) and co-PI (SD) of the study.

## References

[1] Deprez PH (2014). Endoscopic submucosal dissection (ESD): still a matter for debate or a gold standard technique in both Western and eastern countries?. Endosc Int Open.

[2] Kumarasinghe MP, Brown I, Raftopoulos S, Bourke MJ, Charlton A, de Boer WB, Eckstein R, Epari K, Gill AJ, Lam AK, Price T, Streutker C, Lauwers GY (2014). Standardised reporting protocol for endoscopic resection for Barrett oesophagus associated neoplasia: expert consensus recommendations. Pathology.

[3] Kumarasinghe MP, Bourke MJ, Brown I, Draganov PV, McLeod D, Streutker C, Raftopoulos S, Ushiku T, Lauwers GY (2020). Pathological assessment of endoscopic resections of the gastrointestinal tract: a comprehensive clinicopathologic review. Mod Pathol.

[4] Pimentel-Nunes P, Dinis-Ribeiro M, Ponchon T, Repici A, Vieth M, De Ceglie A, Amato A, Berr F, Bhandari P, Bialek A (2015). Endoscopic submucosal dissection: European Society of Gastrointestinal Endoscopy (ESGE) guideline. Endoscopy.

[5] Vieth M, Stolte M (2005). Pathology of early upper GI cancers. Best Pract Res Clin Gastroenterol.

[6] Lugli A, Kirsch R, Ajioka Y, Bosman F, Cathomas G, Dawson H, El Zimaity H, Fléjou JF, Hansen TP, Hartmann A (2017). Recommendations for reporting tumor budding in colorectal cancer based on the international tumor budding consensus conference (ITBCC) 2016. Mod Pathol.

[7] Abraham SC, Krasinskas AM, Correa AM, Hofstetter WL, Ajani JA, Swisher SG, Wu TT (2007). Duplication of the muscularis mucosae in Barrett esophagus: an underrecognized feature and its implication for staging of adenocarcinoma. Am J Surg Pathol.

[8] Sepesi B, Watson TJ, Zhou D, Polomsky M, Litle VR, Jones CE, Raymond DP, Hu R, Qiu X, Peters JH (2010). Are endoscopic therapies appropriate for superficial submucosal esophageal adenocarcinoma? An analysis of esophagectomy specimens. J Am Coll Surg.

[9] Estrella JS, Hofstetter WL, Correa AM, Swisher SG, Ajani JA, Lee JH, Bhutani MS, Abraham SC, Rashid A, Maru DM (2011). Duplicated muscularis mucosae invasion has similar risk of lymph node metastasis and recurrence-free survival as intramucosal esophageal adenocarcinoma. Am J Surg Pathol.

[10] Gockel I, Sgourakis G, Lyros O, Polotzek U, Schimanski CC, Lang H, Hoppo T, Jobe BA (2011). Risk of lymph node metastasis in submucosal esophageal cancer: a review of surgically resected patients. Expert Rev Gastroenterol Hepatol.

[11] Davison JM, Landau MS, Luketich JD, McGrath KM, Foxwell TJ, Landsittel DP, Gibson MK, Nason KS (2016). A Model Based on Pathologic Features of Superficial Esophageal Adenocarcinoma Complements Clinical Node Staging in Determining Risk of Metastasis to Lymph Nodes. Clin Gastroenterol Hepatol.

[12] Peters FP, Brakenhoff KP, Curvers WL, Rosmolen WD, Fockens P, ten Kate FJ, Krishnadath KK, Bergman JJ (2008). Histologic evaluation of resection specimens obtained at 293 endoscopic resections in Barrett's esophagus. Gastrointest Endosc.

[13] Lewis JT, Wang KK, Abraham SC (2008). Muscularis mucosae duplication and the musculo-fibrous anomaly in endoscopic mucosal resections for Barrett esophagus: implications for staging of adenocarcinoma. Am J Surg Pathol.

[14] Alvarez Herrero L, Pouw RE, van Vilsteren FG, ten Kate FJ, Visser M, van Berge Henegouwen MI, Weusten BL, Bergman JJ (2010). Risk of lymph node metastasis associated with deeper invasion by early adenocarcinoma of the esophagus and cardia: study based on endoscopic resection specimens. Endoscopy.

[15] Hoteya S, Matsui A, Iizuka T, Kikuchi D, Yamada A, Yamashita S, Furuhata T, Domon K, Nakamura M, Mitani T, Ogawa O, Kasie M (2013). Comparison of the clinicopathological characteristics and results of endoscopic submucosal dissection for esophagogastric junction and non-junctional cancers. Digestion.

[16] Osumi H, Fujisaki J, Omae M, Shimizu T, Yoshio T, Ishiyama A, Hirasawa T, Tsuchida T, Yamamoto Y, Kawachi H, Yamamoto N, Igarashi M (2017). Clinicopathological features of Siewert type II adenocarcinoma: comparison of gastric cardia adenocarcinoma and Barrett's esophageal adenocarcinoma following endoscopic submucosal dissection. Gastric Cancer.

[17] Imai T (1954). The growth of human carcinoma: a morphological analysis. Fukuoka Igaku Zasshi.

[18] Koelzer VH, Langer R, Zlobec I, Lugli A (2014). Tumor budding in upper gastrointestinal carcinomas. Front Oncol.

[19] Berg KB, Schaeffer DF (2018). Tumor budding as a standardized parameter in gastrointestinal carcinomas: more than just the colon. Mod Pathol.

[20] Brown M, Sillah K, Griffiths EA, Swindell R, West CM, Page RD, Welch IM, Pritchard SA (2010). Tumour budding and a low host inflammatory response are associated with a poor prognosis in oesophageal and gastro-oesophageal junction cancers. Histopathology.

[21] Landau MS, Hastings SM, Foxwell TJ, Luketich JD, Nason KS, Davison JM (2014). Tumor budding is associated with an increased risk of lymph node metastasis and poor prognosis in superficial esophageal adenocarcinoma. Mod Pathol.

[22] Thies S, Guldener L, Slotta-Huspenina J, Zlobec I, Koelzer VH, Lugli A, Kröll D, Seiler CA, Feith M, Langer R (2016). Impact of peritumoral and intratumoral budding in esophageal adenocarcinomas. Hum Pathol.

[23] Ishihara R, Oyama T, Abe S, Takahashi H, Ono H, Fujisaki J, Kaise M, Goda K, Kawada K, Koike T, Takeuchi M, Matsuda R, Hirasawa D, Yamada M, Kodaira J, Tanaka M, Omae M, Matsui A, Kanesaka T, Takahashi A, Hirooka S, Saito M, Tsuji Y, Maeda Y, Yamashita H, Oda I, Tomita Y, Matsunaga T, Terai S, Ozawa S, Kawano T, Seto Y (2017). Risk of metastasis in adenocarcinoma of the esophagus: a multicenter retrospective study in a Japanese population. J Gastroenterol.

[24] Lam AK KM: Adenocarcinoma of the esophagus and esophagogastric junction NOS. In: WHO classification of Tumors, Digestive System. 5th edition edn. Edited by Lokuhetty D WV, Watanabe R, Cree IA: Lyon: International Agency for Research on Cancer; 2019: 38–47.

[25] Lauwers GY, Ban S, Mino M, Ota S, Matsumoto T, Arai S, Chan HH, Brugge WR, Shimizu M (2004). Endoscopic mucosal resection for gastric epithelial neoplasms: a study of 39 cases with emphasis on the evaluation of specimens and recommendations for optimal pathologic analysis. Mod Pathol.

[26] Conio M, Repici A, Cestari R, Blanchi S, Lapertosa G, Missale G, Della Casa D, Villanacci V, Calandri PG, Filiberti R (2005). Endoscopic mucosal resection for high-grade dysplasia and intramucosal carcinoma in Barrett's esophagus: an Italian experience. World J Gastroenterol.

[27] Mino-Kenudson M, Brugge WR, Puricelli WP, Nakatsuka LN, Nishioka NS, Zukerberg LR, Misdraji J, Lauwers GY (2005). Management of superficial Barrett's epithelium-related neoplasms by endoscopic mucosal resection: clinicopathologic analysis of 27 cases. Am J Surg Pathol.

[28] Bartel MJ, Wallace TM, Gomez-Esquivel RD, Raimondo M, Wolfsen HC, Woodward TA, Wallace MB (2017). Role of EUS in patients with suspected Barrett's esophagus with high-grade dysplasia or early esophageal adenocarcinoma: impact on endoscopic therapy. Gastrointest Endosc.

[29] Thota PN, Sada A, Sanaka MR, Jang S, Lopez R, Goldblum JR, Liu X, Dumot JA, Vargo J, Zuccarro G (2017). Correlation between endoscopic forceps biopsies and endoscopic mucosal resection with endoscopic ultrasound in patients with Barrett's esophagus with high-grade dysplasia and early cancer. Surg Endosc.

[30] Swager AF, Tearney GJ, Leggett CL, van Oijen MGH, Meijer SL, Weusten BL, Curvers WL, Bergman J (2017). Identification of volumetric laser endomicroscopy features predictive for early neoplasia in Barrett's esophagus using high-quality histological correlation. Gastrointest Endosc.

